# Microbiological-Chemical Sourced Chondroitin Sulfates Protect Neuroblastoma SH-SY5Y Cells against Oxidative Stress and Are Suitable for Hydrogel-Based Controlled Release

**DOI:** 10.3390/antiox10111816

**Published:** 2021-11-16

**Authors:** Emiliano Bedini, Alfonso Iadonisi, Chiara Schiraldi, Laura Colombo, Diego Albani, Paola Petrini, Carmen Giordano, Marta Tunesi

**Affiliations:** 1 Department of Chemical Sciences, University of Naples Federico II, Via Cinthia 4, I-80126 Naples, Italy; ebedini@unina.it (E.B.); iadonisi@unina.it (A.I.); 2 Department of Experimental Medicine, Section of Biotechnology, Medical Histology and Molecular Biology, University of Campania Luigi Vanvitelli, via L. De Crecchio 7, I-80138 Naples, Italy; chiara.schiraldi@unicampania.it; 3 Department of Molecular Biochemistry and Pharmacology, Istituto di Ricerche Farmacologiche Mario Negri IRCCS, via M. Negri 2, I-20156 Milan, Italy; laura.colombo@marionegri.it; 4 Department of Neuroscience, Istituto di Ricerche Farmacologiche Mario Negri IRCCS, via M. Negri 2, I-20156 Milan, Italy; diego.albani@marionegri.it; 5 Department of Chemistry, Materials and Chemical Engineering “G. Natta”, Politecnico di Milano, Piazza L. da Vinci 32, I-20133 Milan, Italy; paola.petrini@polimi.it (P.P.); carmen.giordano@polimi.it (C.G.)

**Keywords:** sulfation pattern, neuroprotection, hydrogen peroxide, DMMB assay, agarose, Carbomer homopolymers, Carbopol 974P NF, steam sterilization, viscoelastic properties, drug loading

## Abstract

Chondroitin sulfates (CS) are a class of sulfated glycosaminoglycans involved in many biological processes. Several studies reported their protective effect against neurodegenerative conditions like Alzheimer’s disease. CS are commonly derived from animal sources, but ethical concerns, the risk of contamination with animal proteins, and the difficulty in controlling the sulfation pattern have prompted research towards non-animal sources. Here we exploited two microbiological-chemical sourced CS (i.e., CS-A,C and CS-A,C,K,L) and Carbopol 974P NF/agarose semi-interpenetrating polymer networks (i.e., P.NaOH.0 and P.Ethanol.0) to set up a release system, and tested the neuroprotective role of released CS against H_2_O_2_-induced oxidative stress. After assessing that our CS (1–100 µM) require a 3 h pre-treatment for neuroprotection with SH-SY5Y cells, we evaluated whether the autoclave type (i.e., N- or B-type) affects hydrogel viscoelastic properties. We selected B-type autoclaves and repeated the study after loading CS (1 or 0.1 mg CS/0.5 mL gel). After loading 1 mg CS/0.5 mL gel, we evaluated CS release up to 7 days by 1,9-dimethylmethylene blue (DMMB) assay and verified the neuroprotective role of CS-A,C (1 µM) in the supernatants. We observed that CS-A,C exhibits a broader neuroprotective effect than CS-A,C,K,L. Moreover, sulfation pattern affects not only neuroprotection, but also drug release.

## 1. Introduction

**Figure 1:**
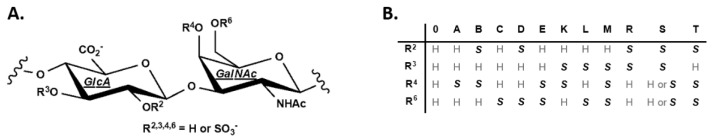
(**A**) Disaccharide-repeating unit of CS, composed of D-glucuronic acid (GlcA) and 2-acetamido-2-deoxy-D-galactosamine (*N*-acetyl-galactosamine, GalNAc) linked together through alternating β-1→3 and β-1→4 glycosidic bonds; (**B**) sulfation patterns in CS from natural sources (***S*** = SO_3_^-^).

Chondroitin sulfates (CS) are a class of glycosaminoglycans (GAGs) widely distributed in both vertebrates and invertebrates, playing key roles in several biological events. Their disaccharide-repeating unit can be sulfated to various extents (Figure 1). Interestingly, in CS sourced from terrestrial animals, sulfate groups are almost exclusively found at positions *C*-4 and/or *C*-6 of *N*-acetyl-galactosamine (GalNAc) units to give the so-called A,C and E subunits; while CS from marine organisms often display additional sulfate groups at *C*-2 and/or *C*-3 of D-glucuronic acid (GlcA) residues [1].

Sulfation pattern depends not only on animal species and tissue, but also on the physio-pathological conditions (aging, inflammation, tumor formation, etc.). Noteworthy, it seems to encode functional information in a plethora of physiological and pathological processes, but only few of them have been unveiled in detail [2].

CS form proteoglycans (CSPGs), an essential component of the extracellular matrix (ECM), to which they confer peculiar properties. In cartilage, the electrostatic charges due to the densely packed sulfate groups provide much of tissue resistance to compression, and loss of CS is a major cause of osteoarthritis [3]. In perineuronal nets (PNNs), the amount of CSPGs is very limited (about 2% of CSPGs in the nervous system [4,5]), but in adults their sulfation pattern differs from that of diffuse ECM [4] and strongly influences the formation and binding properties of PNNs [6,7]. PNNs show protective effects against tau pathology [8], oxidative stress [9], and calcium-dependent excitotoxicity [10]. In Alzheimer’s disease (AD) patients, the number of PNNs is not reduced [11], but a decrease in CS is detectable [12], suggesting their neuroprotective effect. CS oligosaccharides may bind Aβ fibrils by electrostatic interactions. This promotes the self-assembly of toxic oligomeric precursors into harmless fibrils, prevents their interaction with cells and mitochondrial membranes [13,14], and blocks the activation of microglial cells [15]. In particular, low molecular weight CS reduce cell apoptosis by enhancing the activity of antioxidant enzymes [15]. Protective effects of CS against advanced glycation end product-induced toxicity, a condition found in diabetes, cardiovascular and neurological diseases (e.g., AD, Parkinson’s disease) were described [13], together with anti-inflammatory, anti-atherosclerotic, immunoregulatory, and antioxidant functions [16,17]. In particular, CS antioxidant action against H_2_O_2_-induced oxidative stress is mediated by HO-1 enzyme [18].

Pharmaceutical preparations and dietary supplements exploit CS to treat or prevent some diseases [19], the main one being osteoarthritis (e.g., Condrosulf^®^, Condrosulf Unidie, Matrix^®^ [20]). CS from animal sources pose some concerns, such as ethical issues, the risk of contamination with animal proteins, and the difficulty in controlling the sulfation pattern. For instance, oversulfated CS in heparin lots were identified as the etiological agent of severe anaphylactic reactions after intravenous administration [21]. For these reasons, a different access to CS has attracted considerable efforts in the last two decades [22]. Microbial cell factories represent a promising approach. They can be engineered to produce CS directly [23,24] or combined with tailored chemical procedures aimed at decorating unsulfated chondroitin with well-defined sulfation patterns [25]. Noteworthy, the latter strategy leads to CS polysaccharides with biological responses comparable to animal sourced CS with the same sulfation degree [26,27].

For the treatment of osteoarthritis, CS are sometimes coupled with glucosamine and orally administered (usually 800–1200 mg/day). The absorption from the gastrointestinal tract depends on molecular weight, density charge, and hydrophilicity [28]. Since a relevant fraction reaches cartilage and other GAG-rich tissues [20], bioavailability is limited to 10–20% [29]. To reduce the dose and improve local effects, intra-articular injections of CS-loaded hydrogels were proposed [30,31], but the lack of sustained release due to joint fluids and the risk of infections were the major disadvantages. Despite the possible potential benefits, CS-loaded delivery systems for brain applications have not been described yet.

Hydrogels are widely exploited for drug delivery. For example, Carbopols^®^ are mucoadhesive, high molecular weight, crosslinked poyacrylic acids extensively used to deliver hydrophobic molecules [32], and in ophthalmic [33], topical [34], vaginal [35], buccal [36], intestinal [37], and nasal [38] preparations. Among them, Carbopol 974P NF is crosslinked with allyl ethers of pentaerythritol in ethyl acetate and used in several FDA-approved drugs [39]. Among hydrogels based on natural polymers, agarose can be exploited for drug release because of its low cost, gelling ability, thermoreversibility and viscoelastic properties tunable with concentration [40]. Hydrogel preparation is simple and does not require harsh reagents. Agarose dissolves in boiling water and gels after cooling below 45 °C because of extensive hydrogen bonding between chains, without additional cross-linking steps. The gelling temperature varies with molecular weight, concentration, chemical modification of side groups, and presence of destructuring salts [41]. The sol-gel transition allows for the homogeneous loading and distribution of bioactive molecules in the sol phase, without any risk of degradation for the thermolabile ones. A further advantage of thermoreversibility is the possibility to inject the material in situ as a viscous fluid, thus limiting the invasiveness of the administration.

In a previous study, we combined Carbopol 974P NF and agarose and exploited the final semi-interpenetrating polymer networks (semi-iPNs) for resveratrol release [42], a natural polyphenol with poor bioavailability but high therapeutic interest [43], also against brain disorders. As semi-IPNs, Carbopol 974P NF/agarose hydrogels join two independent and physically interlocked networks and combine the properties of both components in a tunable manner [44]. For instance, semi-IPNs exhibit more rapid kinetic response rates to pH or temperature than single network gels [45]. Another peculiar advantage of Carbopol 974P NF/agarose hydrogels relies on their preparation by autoclaving. Due to its fastness and effectiveness, autoclaving is the easiest sterilization procedure approved by the US Food and Drug Administration [46]. In the case of Carbopol 974P NF/agarose hydrogels, it allows for simultaneously heating up agarose and sterilizing the final product, an essential requirement for biomedical applications.

Starting from microbiological-chemical sourced CS and Carbopol 974P NF/agarose semi-iPNs, here we report the setting-up of injectable CS-loaded hydrogels suitable to deliver CS also to brain tissues, and we demonstrate the neuroprotective effect of released CS against H_2_O_2_-induced oxidative stress in a neuroblastoma cell line.

## 2. Materials and Methods

Carbopol^®^ 974P NF was gifted by Lubrizol Corporation (Wickliffe, OH, USA). Plasticware was purchased from Corning Costar Corp (Corning, NY, USA), while Ultra-Pure^TM^ agarose and reagents for cell culture were obtained from Thermo Fisher Scientific (Waltham, MA, USA). If not differently stated, reagents were from Sigma-Aldrich (St. Louis, MO, USA).

### 2.1. Synthesis and Characterization of CS Polysaccharides

The CS polysaccharides used in this study are composed of A,C and A,C,K,L subunits (CS-A,C and CS-A,C,K,L, respectively; Figure 1) randomly interspersed along the polysaccharide chain. They were obtained by semi-synthesis from unsulfated chondroitin, which was produced by fed-batch fermentation of recombinant *Escherichia coli* K4 and purified using ultrafiltration, mild hydrolysis, and ethanol precipitation [47]. The detailed procedures for the semi-syntheses of CS-A,C and CS-A,C,K,L as well as their physical-chemical characterization by 2D-NMR and high-performance size exclusion chromatography combined with a triple detector array (HP-SEC-TDA) are described in previous works [48,49].

### 2.2. Cell Culture

SH-SY5Y cells (ATCC^®^ code CRL-2266™) were cultured at 37 °C, 5% CO_2_ in high glucose Dulbecco’s modified Eagle’s medium (DMEM) supplemented with 10% *v*/*v* fetal bovine serum, 2 mM L-glutamine, 100 U/mL penicillin, 100 µg/mL streptomycin sulfate. Medium was replaced every 2–3 days and cells were passaged twice a week.

### 2.3. Neuroprotective Properties of CS Polysaccharides

CS-A,C and CS-A,C,K,L were dissolved in sterile water for injection (Eurospital, Trieste, Italy) to a concentration of 1 mM, diluted with culture medium to 100, 10, and 1 µM, and sterilized with syringe filters with a 0.22 µm pore size.

For all cell experiments, 10^5^ SH-SY5Y cells/cm^2^ were plated in 96-well microplates. In all the steps, the working volume was 100 µL/well.

To investigate whether CS polysaccharides exhibit cytotoxic effects, one day after plating cells were exposed to CS (1, 10, 100 µM). After 27 h, cell viability was assessed by MTS assay (Promega, Madison, WI, USA). Medium was replaced with fresh culture medium supplemented with 10% *v*/*v* MTS. After 3 h incubation at 37 °C, 5% CO_2_, sample absorbance was measured at 490 nm with a spectrophotometer (Infinite M200 PRO, Tecan, Männedorf, Switzerland). As controls, SH-SY5Y cells were cultured in standard medium.

To induce oxidative stress and set up the cytotoxicity model, one day after plating cells were exposed to H_2_O_2_ (50, 75, 100 µM). After 24 h, cell viability was assessed by MTS assay, as described. As controls, SH-SY5Y cells were cultured in standard medium.

To investigate whether CS polysaccharides exhibit neuroprotective properties, one day after plating cells were incubated with CS (1, 10, 100 µM). After 3 h, 75 µM H_2_O_2_ was added. After 24 h, cell viability was evaluated by MTS assay, as described. As controls, SH-SY5Y cells were cultured in standard medium.

### 2.4. Preparation and Characterization of Carbomer/Agarose Semi-IPNs

Carbopol^®^ 974P NF (0.73% *w*/*v*) was dissolved in phosphate buffered saline (PBS), then the neutralizing agent (NaOH for P.NaOH.0 gels, triethanolamine for P.Ethanol.0 gels; 7.2 mmol/g Carbomer) and agarose (0.5 *g*/*g* Carbomer) were added and the suspensions were autoclaved. Gelation was achieved during cooling. Additional details about the preparation of P.NaOH.0 and P.Ethanol.0 gels as well as their viscoelastic properties and cytocompatibility with fibroblast-like and neuronal-like cells are described in a previous work [42].

### 2.5. Effect of the Sterilization Process on the Viscoelastic Properties of Carbomer/Agarose Semi-IPNs

Steam sterilization relies on the injection of moist heat under high pressure and temperature in a closed chamber. It is cost-effective, fast, rapidly microbicidal, and reliable [50]. According to the process of removing air to allow for the entrance of saturated steam and the type of load that can be sterilized, autoclaves are classified into N, S, and B-types. N-types are non-vacuum autoclaves, where air is passively displaced by steam. They are common as bench-top sterilizers, but because of the absence of a pump, they are suitable only for unwrapped solid objects, without pores or cavities. S-type steam sterilizers are suitable only for the types of load specified by the manufacturer, while B-type autoclaves are equipped with a vacuum pump and exhibit a fractionated pre-vacuum. They are the more advanced ones and are suitable for every type of load, eventually wrapped [51,52]. However, N-type steam sterilizers are commonly used to treat laboratory media, water, pharmaceutical products, nonporous objects whose surface is directly exposed to steam, and regulated medical waste [50].

To investigate whether the sterilization process (i.e., the presence or absence of vacuum during autoclaving) affects hydrogel viscoelastic properties, the suspensions for P.NaOH.0 and P.Ethanol.0 gels were sterilized at 121 °C for 20 min in an N- (760, Asal s.r.l., Cernusco sul Naviglio, Milan, Italy) or B-type (NT80, Golmar, Milan, Italy) bench-top autoclave. In both conditions, the maximum volume to be sterilized was 15 mL, and it was placed in an unpacked 50 mL centrifuge tube. After sterilization, the suspensions were poured into 100 mm Petri dishes to obtain a thickness of 3 mm. After gelation, samples were stored overnight at 4 °C.

Rheological analyses were run at 25 °C with an AR 1500ex rheometer (TA Instruments, New Castle, DE, USA) equipped with parallel-plate geometry (20 mm diameter, 2000 µm working gap). To identify the linear viscoelastic region, amplitude sweeps were performed while varying the stress from 0.1 to 100 Pa. Storage (G′), loss (G″) moduli, and complex viscosity were measured as a function of frequency. Frequency sweeps were run at 1% strain while varying the frequency from 1 to 10 Hz. G′ and G″ were also evaluated as a function of temperature. Temperature ramps were performed in the range 20–100 °C with a heating ramp of 5 °C/min.

### 2.6. Effect of CS Polysaccharide Loading on the Viscoelastic Properties of Carbomer/Agarose Semi-IPNs

CS-A,C and CS-A,C,K,L were dissolved in sterile water for injection to a concentration of 1 mM and sterilized with syringe filters with a 0.22 µm pore size.

P.NaOH.0 or P.Ethanol.0 suspensions were autoclaved in the B-type steam sterilizer. During cooling and before gelation, at about 37 °C, CS polysaccharides were mixed to the polymer solutions to a final concentration of 10 or 100 µM (i.e., 5 or 50 µL CS/0.5 mL gel). To investigate whether CS loading affects hydrogel viscoelastic properties, frequency sweeps and temperature ramps were run as described in Section 2.5. As control, the analyses were performed for semi-IPNs loaded with distilled water (5 or 50 µL water/0.5 mL gel).

### 2.7. CS Polysaccharide Release from Carbomer/Agarose Semi-IPNs

Samples (50 µL CS/0.5 mL gel, that is 1 mg CS) were prepared in custom-made cylindrical molds (inner diameter: 11.05 mm) in 12-well plates. After gelation, the molds were removed. Samples were covered with 2.5 mL PBS and placed on an orbital shaker at 25 °C. After 1, 4, 24, 48, 72, 96, and 168 h, the supernatants were collected and replaced with 2.5 mL fresh PBS. CS in the supernatants were evaluated by 1,9-dimethylmethylene blue (DMMB) assay [53]. To avoid the precipitation of the dye–substrate complex, 1 part *v*/*v* supernatant was gently mixed to 25 parts *v*/*v* assay solution (pH 3.0, 1 L), prepared by dissolving 16 mg DMMB in water, in the presence of 3.04 g glycine, 2.37 g NaCl, and 95 mL HCl 0.1 M. Within 1 min, sample absorbance was measured at 525 nm with a UV-visible spectrophotometer (Lambda 19, PerkinElmer, Waltham, MA, USA). For a robust calibration line, we considered three replicates of six concentration values in the range 0.5–5 µg. As control, the analyses were run on supernatants from samples loaded with distilled water (50 µL water/0.5 mL gel), whose mean absorbance was subtracted from that of supernatants from CS-loaded hydrogels.

Since in previous tests CS-A,C had exhibited the broader neuroprotective effects, we assessed whether hydrogel degradation products affect the cytocompatibility of released CS-A,C and whether released CS-A,C retain their neuroprotective properties.

To reach the first goal, 10^5^ SH-SY5Y cells/cm^2^ were plated in 96-well microplates. One day after plating, cells were exposed to 1 µM CS, prepared by diluting the supernatants (up to 1 part *v*/*v*) with 2X culture medium (1 part *v*/*v*). After 27 h, cell viability was assessed by MTS assay. As controls, SH-SY5Y cells were cultured in standard culture medium or freshly dissolved 1 µM CS.

To reach the second goal, 10^5^ SH-SY5Y cells/cm^2^ were plated in 96-well microplates. One day after plating, medium was replaced with supernatants containing 1 µM CS, as described. After 3 h, 75 µM H_2_O_2_ was added. After 24h, cell viability was evaluated by MTS assay, as described. As controls, SH-SY5Y cells were cultured in standard culture medium, eventually supplemented with freshly dissolved 1 µM CS.

In all the steps of both investigations, the working volume was 100 µL/well.

### 2.8. Statistical Analysis

Experiments were run at least in triplicate. *N* refers to the number of independent repetitions. Results were reported as mean ± standard deviation (SD) and analyzed with GraphPad Prism (GraphPad Software, San Diego, CA, USA), release 8. After assessing the normality of data, differences among the groups were analyzed by one-way ANOVA followed by Dunnett’s multiple comparisons test. Differences were considered as statistically significant when *p*-value < 0.05.

## 3. Results

### 3.1. Neuroprotective Properties of CS Polysaccharides

**Figure 2:**
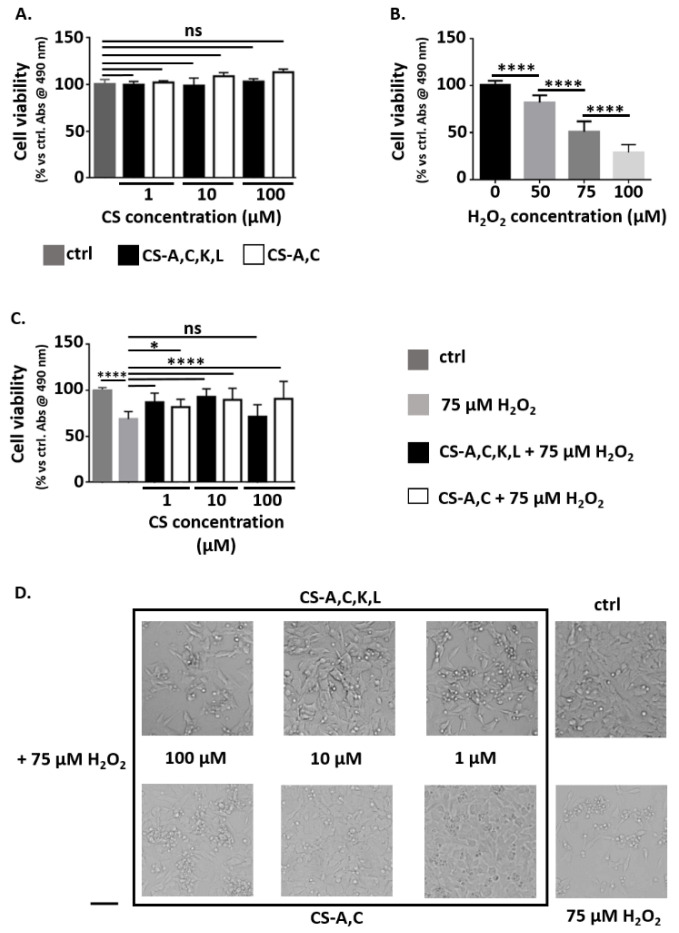
(**A**) Viability of SH-SY5Y cells after 27 h incubation with CS (1, 10, 100 µM) with respect to controls in standard medium (ctrl). Results from MTS assay. *N* = 3, 4 samples each. (**B**) Viability of SH-SY5Y cells after 24 h incubation with H_2_O_2_ (50, 75, 100 µM) with respect to controls in standard medium (ctrl). Results from MTS assay. *N* = 3, 6 samples each. (**C**) Viability of SH-SY5Y cells after 24 h incubation with 75 µM H_2_O_2_ in the presence of CS (1, 10, 100 µM) with respect to controls in standard medium (ctrl). Results from MTS assay. *N* = 4, 4 samples each. (**D**) Analysis by optical microscopy of SH-SY5Y cell morphology after 24 h incubation in standard medium (ctrl), eventually with 75 µM H_2_O_2_ and CS polysaccharides (1, 10, 100 µM). Scale bar: 10 µm. Statistical analysis was performed with one-way ANOVA followed by Dunnett’s multiple comparisons test. ns: *p*-value > 0.05; * *p*-value < 0.05; **** *p*-value < 0.0001.

Independently on concentration and for incubation with both CS-A,C and CS-A,C,K,L, cell viability was comparable to controls (ns, *p*-value > 0.05, Figure 2A). In all the conditions, optical microscopy showed elongated cell morphology, with numerous cytoplasmic protrusions (data not reported). These results indicated the absence of cytotoxic effects for CS polysaccharides at 1, 10, and 100 µM.

Hydrogen peroxide is a common trigger of oxidative stress [54] (Figure 2B). For incubation with 50 or 75 μM H_2_O_2_, optical microscopy revealed rounded or suspended cells, but most of them were still adherent to the microplate. For 100 μM H_2_O_2_, the presence of rounded or suspended cells increased. All the differences among the concentrations were significant (****, *p*-value < 0.0001). Cell viability was reduced by 20% for incubation with 50 μM H_2_O_2_, by 50% for incubation with 75 μM, and by 70% for incubation with 100 μM. According to these results, we selected 75 μM H_2_O_2_ as oxidative stressor to test the neuroprotective effect of CS-A,C and CS-A,C,K,L polysaccharides.

Independently on CS-A,C concentration, a 3 h pre-treatment before adding H_2_O_2_ was sufficient to maintain cell viability at values comparable with controls (ns, *p*-value > 0.05) (Figure 2C). Optical microscopy showed cells adherent to the microplate, with an elongated shape and numerous long processes (Figure 2D). For CS-A,C,K,L, the pre-treatment was not effective at a concentration of 100 μM. In this condition, cell viability was comparable to that of cells incubated with 75 μM H_2_O_2_ (*p*-value > 0.05, Figure 2C). These results suggested that CS-A,C has broader neuroprotective effects. However, we found that when H_2_O_2_ reduced cell viability by 22–32% with respect to controls, also 100 μM CS-A,C,K,L maintained cell viability at values comparable with controls (ns, *p*-value > 0.05, data not shown).

### 3.2. Effect of Sterilization Process on the Viscoelastic Properties of Carbomer/Agarose Semi-IPNs

**Figure 3:**
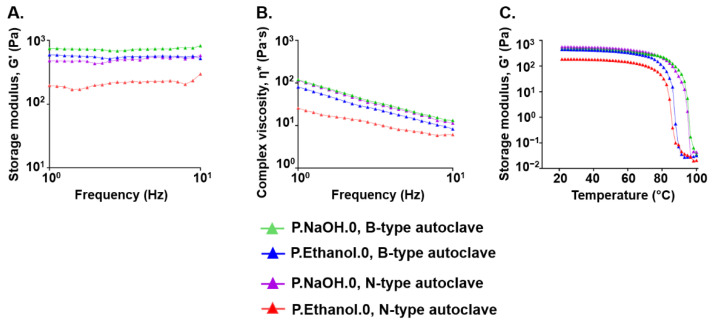
Frequency sweep tests: (**A**) storage modulus (G′) and (**B**) complex viscosity as a function of frequency; (**C**) temperature ramp tests: storage modulus (G′) as a function of temperature for suspensions sterilized in B- or N-type autoclaves. For all the investigations, *N* = 3, 1 sample each.

In agreement with a previous study where the suspensions for P.NaOH.0 and P.Ethanol.0 were heated in an N-type steam sterilizer [42], at all the frequencies tested storage modulus and complex viscosity were higher for P.NaOH.0 than P.Ethanol.0, contributing to the solid-like behavior (Figure 3A,B). A more organized and stable network was confirmed by the increase of the onset temperature over 80 °C to undergo a transition from gel to liquid state (Figure 3C). When testing semi-IPNs prepared with B-type autoclaves, rheological curves exhibited the same trends observed when the suspensions were sterilized in N-type steam sterilizers, but differences between P.NaOH.0 and P.Ethanol.0 gels were reduced. The sterilization with B-type autoclaves produce semi-IPNs with higher solid contribution. For this reason, we used B-type autoclaves for further experiments.

### 3.3. Effect of CS Polysaccharide Loading on the Viscoelastic Properties of Carbomer/Agarose Semi-IPNs

**Figure 4:**
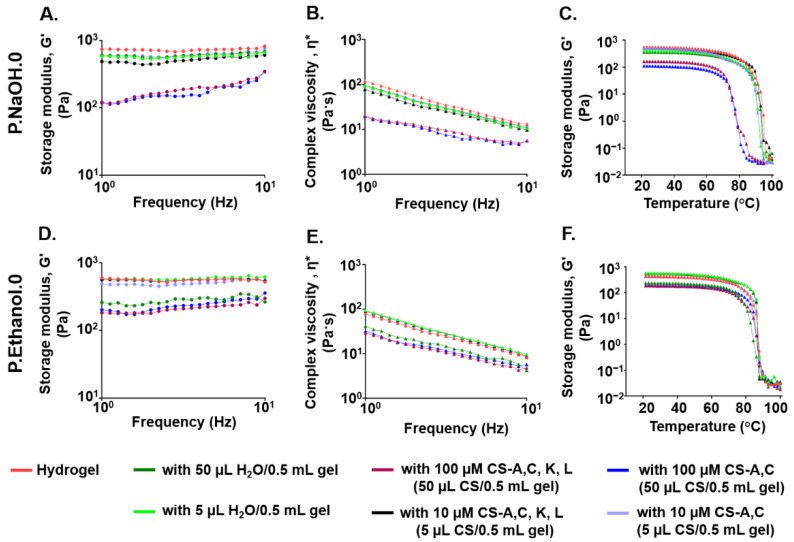
Frequency sweep tests: (**A**,**D**) storage modulus (G′) and (**B**,**E**) complex viscosity as a function of frequency; (**C**,**F**) temperature ramp tests: storage modulus (G′) as a function of temperature for P.NaOH.0 and P.Ethanol.0, respectively. Hydrogels were produced with B-type autoclaves and loaded with distilled water or CS polysaccharides. For all the investigations, *N* = 3, 1 sample each.

CS altered the viscoelastic properties of both hydrogels, with a dependence on the loading dose. When loading 100 µM CS polysaccharides, G′ was reduced with respect to the initial condition at all the frequencies tested, suggesting that the drug partially interferes with network formation. G′ was dependent on frequency, but the increase of G′ with frequency was more apparent for P.NaOH.0. Independently on CS, at the end of the measurements the storage moduli of P.NaOH.0 and P.Ethanol.0 were comparable. When loading 10 µM CS, G′ was independent on frequency and it was not considerably reduced with respect to the initial condition. For P.NaOH.0, a reduction of G′ was not found when loading 50 µL water, suggesting that the decrease was not due to hydrogel dilution while loading, but to the presence of CS polysaccharides. On the contrary, for P.Ethanol.0 a reduction of G′ was also observed, suggesting that hydrogel dilution could be involved. For both gels, no significant variations of G′ were detected when loading 5 µL water/sample (Figure 4A,D).

When loading CS, the shear thinning behavior was maintained. When loading 100 µM CS polysaccharides, at low frequencies higher complex viscosities were measured for P.Ethanol.0 with respect to P.NaOH.0 (Figure 4B,E).

In the range of physiological temperatures, for both gels G′ profile was independent on temperature. When loading 100 µM CS, P.Ethanol.0 network was more stable in temperature with respect to P.NaOH.0, as indicated by the greater onset point (Figure 4C,F). These results support the hypothesis of a more organized network, thus requiring a higher amount of energy to be disrupted. When loading 100 µM CS-A,C in P.NaOH.0 or in P.Ethanol.0, the onset points occurred at 72.8 and 82.7 °C, respectively (mean values). When loading 100 µM CS-A,C, K, L in P.NaOH.0 or in P.Ethanol.0, the onset points occurred at lower temperatures (69.0 and 80.8 °C, respectively, mean values). These results support the hypothesis of a possible hindering of network formation due to the interaction with CS.

### 3.4. CS Polysaccharide Release from Carbomer/Agarose Semi-IPNs

**Figure 5:**
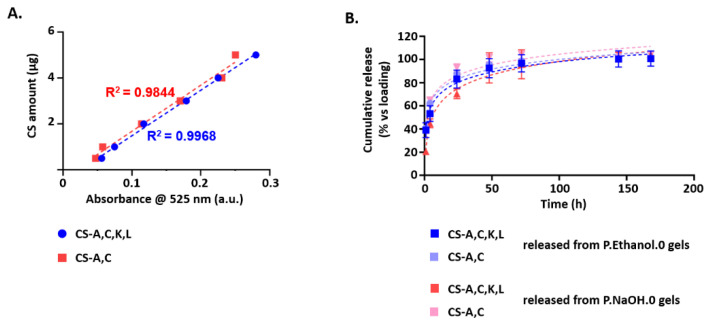
(**A**) Examples of the calibration lines to estimate the amount of CS-A,C and CS-A,C,K,L released from P.Ethanol.0 or P.NaOH.0 gels. *N* = 3, 1 sample each to be diluted to obtain the 6 solutions to be tested; (**B**) release profiles of CS-A,C and CS-A,C,K,L in PBS. Results were reported with respect to the initial loading (1 mg CS/0.5 mL gel). *N* = 4, 1 sample each.

The release of CS polysaccharides was evaluated by DMMB assay, whose sensitivity is less than 4 μg/mL of CS [55]. At each time point, the amount of CS-A,C, K, L or CS-A,C in the supernatants was estimated with a calibration line based on six standards (examples are shown in Figure 5A). Both hydrogels were suitable for a prolonged release of CS polysaccharides (Figure 5B). CS-A,C, K, L were totally released from P.Ethanol.0 gels in 144 h; while about 85% of the loaded mass was released from P.NaOH.0 gels in 7 days. CS-A,C were totally released from P.NaOH.0 gels in 48–72 h, and from P.Ethanol.0 gels in 96 h.

To determine the release kinetics, release profiles were fitted with various release kinetic models. For all the conditions, the best results were achieved with the Korsmeyer–Peppas model ($Kt^{n}=\frac{Mt}{M\infty })$, where $\frac{Mt}{M\infty }\;$represents the fraction of CS released at time t, K is the release rate constant, and n is the diffusional exponent [56]. By linear regression analysis, we calculated that n exponent did not exceed 0.31, with the corresponding values of the correlation coefficients (R^2^) ranging from 0.9005 to 0.9659. The values of n indicate that the release mechanism of CS from P.NaOH.0 and P.Ethanol.0 is diffusion-controlled.

After collection, the supernatants containing CS-A,C were tested with SH-SY5Y cells for cytocompatibility and neuroprotection against H_2_O_2_-induced oxidative stress. As a proof of concept, we report the results for 1 µM CS-A,C. The analyses were performed for the supernatants collected up to 48 h. For longer time points, it was not possible to maintain the selected dilution ratio.

**Figure 6:**
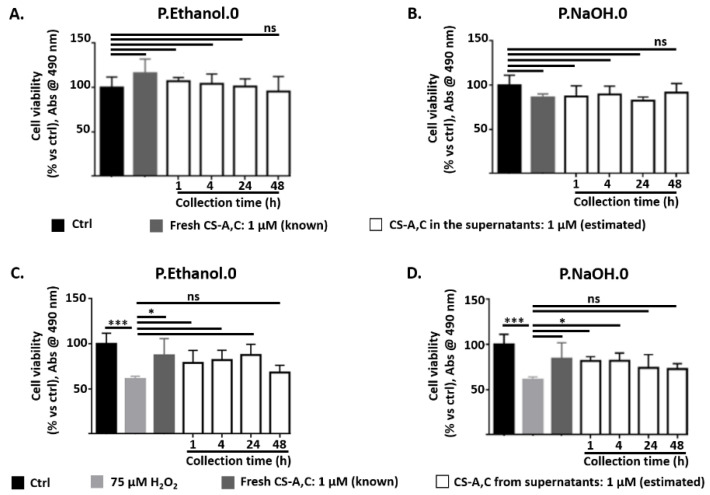
(**A**,**B**) SH-SY5Y cell viability after 27 h incubation with the supernatants containing 1 µM CS-A,C. Supernatants were collected after 1, 4, 24, 48 h from P.Ethanol.0 or P.NaOH.0 gels and eventually diluted to 1 µM; (**C**,**D**) SH-SY5Y cell viability after 24 h incubation with 75 µM H_2_O_2_ in the presence of the supernatants containing 1 µM CS-A,C. Supernatants were collected after 1, 4, 24, 48 h from P.Ethanol.0 or P.NaOH.0 gels and eventually diluted to 1 µM. In both investigations, SH-SY5Y cells were also cultured in standard medium and in freshly dissolved 1 µM CS-A,C. Results from MTS assay. *N* = 4. All the supernatants analyzed in Figure 5B were tested, 1 sample each. Statistical analysis was performed with one-way ANOVA followed by Dunnett’s multiple comparisons test. Ns: *p*-value > 0.05; * *p*-value < 0.05; *** *p*-value < 0.001.

For both P.Ethanol.0 and P.NaOH.0, hydrogel degradation products did not affect the cytocompatibility of released CS (ns, *p*-value > 0.05, Figure 6A,B, respectively).

CS-A,C from P.Ethanol.0 supernatants protected SH-SY5Y cells from oxidative stress at all the time points, except at 48 h (ns, *p*-value > 0.05). At this time point, cell viability was comparable to the case of incubation with 75 µM H_2_O_2_ (ns, *p*-value > 0.05), while at the previous ones it was comparable to that of controls in standard medium or incubated with freshly dissolved CS-A,C (*, *p*-value < 0.05). In the presence of 1 µM CS-A,C from P.NaOH.0 supernatants, cell viability recovered to values greater than that measured for the H_2_O_2_ group only at 1 and 4 h (*, *p*-value < 0.05). In particular, at these time points cell viability was comparable to that measured for both controls (ns, *p*-value > 0.05). For the supernatants collected at 24 and 48 h, the mean values of cell viability were greater than that measured for the H_2_O_2_ group, but the difference was not statistically significant (ns, *p*-value > 0.05). Finally, we excluded the involvement of hydrogel degradation products in neuroprotection (data not shown).

## 4. Discussion

Our study moved forward from the potential of CS in a range of biomedical applications and against oxidative stress. In biomedical applications, they have been proposed as coatings for dental, bone, and vascular implants [57,58,59], as building blocks in scaffolds or hydrogels for cell/drug release [60], and as biosensors [61], due to their abundance and pivotal functions in the human body. They can counteract oxidative stress, a risk factor of several neurodegenerative conditions, such as AD, schizophrenia, bipolar disorders, and Parkinson’s disease [62,63,64,65]. The potential of CS was also highlighted by therapeutic interventions targeting neural ECM and PNNs. For instance, the treatment with chondroitinase ABC contributes to axonal regeneration and functional recovery after acute spinal cord injury [66], and to memory improvement in models of neurodegeneration and neurotoxicity. However, it lacks selectivity, because chondroitinase ABC degrades not only CS in CSPGs, but also dermatan sulfate and slowly in hyaluronate [67].

Despite the promising results, the translation of CS-based products to clinical practice remains challenging. The animal origin of CS, the batch-to-batch variations leading to differences in molecular weight and degree of sulfation, and the risk of contamination are among the main reasons [19]. To overcome these drawbacks, we produced structurally defined CS polysaccharides by chemical modification of microbial-sourced unsulfated chondroitin [68] and evaluated their neuroprotective effect in a H_2_O_2_-induced model of oxidative stress. A similar research question was also investigated by Cañas et al. [18], who tested commercial CS from animal origin. Compared to their work, our CS showed interesting advantages: total neuroprotection was achieved after a shorter pre-treatment time (3 h vs. 24 h) before exposure to H_2_O_2_, and for lower CS concentrations (1–100 µM vs. 600–1000 µM). The latter result has positive implications for the aspects related to the development of a delivery system: if our CS polysaccharides ensure neuroprotection at lower concentrations with respect to commercial ones, a lower loading dose is required, without or with minimal variation of the hydrogel viscoelastic properties. As a further support of its biological relevance, we also found that sulfation pattern affects neuroprotection, with CS-A,C exhibiting a broader neuroprotective effect than CS-A,C,K,L.

Low molecular weight GAGs (i.e., with an average molecular weight of 2.0 ± 0.2 kDa) can cross the blood–brain barrier and diffuse in the brain and cerebrospinal fluid [69]. The ability with which a molecule crosses the blood–brain barrier is influenced by its molecular weight. Our CS are easily soluble in water and show an average molecular weight of 17 kDa [48]. These properties disadvantage CS diffusion across the BBB. For this reason, we focused on the development of a delivery system to exploit their potential benefits and extend their bioavailability. To speed up a potential clinical translation, we used Carbopol^®^ 974P NF, a Carbomer homopolymer extensively used in pharmaceutical formulations.

In a previous study [42], we demonstrated that our gels are successfully prepared by autoclaving. However, since sterilization can affect the properties of the final product [70], here we investigated the effect of the type (i.e., N, B) of steam sterilizer. Sterilization in a B-type autoclave does not limit the load type (i.e., hollow or porous products are effectively sterilized) and increases the viscoelastic properties of both P.NaOH.0 and P.Ethanol.0 gels. This is probably due to the more efficient process of air removal from the chamber prior to sterilization. This ensures for a better steam penetration and a greater solvent evaporation while autoclaving.

The characterization of hydrogel viscoelastic properties after loading CS polysaccharides or water suggests that CS interact differently with polymeric matrices. This hypothesis was supported by the results from DMMB assay: CS-A,C is released faster than CS-A,C, K, L; and CS-A,C, K, L is delivered much faster from P.Ethanol.0 than P.NaOH.0. The investigation of drug–polymer interactions (e.g., by FTIR or NMR spectroscopy [71]) was out of the scope of this work, but our results confirmed a structural difference between the two CS polysaccharides and the influence of sulfation pattern also on diffusion.

As a proof of concept to assess whether released CS keep their neuroprotective effect, we focused on CS-A, C because of its broader neuroprotective effect. By DMMB assay, we assessed that CS-A,C is totally released from P.NaOH.0 gels in 48–72 h, and from P.Ethanol.0 gels in 96 h. After incubation with CS sampled at shorter time points (i.e., 1, 4, 24 h), cell viability was comparable to that measured after incubation with the same concentration of freshly dissolved CS. For longer time points (i.e., 48 h for P.Ethanol.0; 24–48 h for P.NaOH.0), the supernatants did not exhibit neuroprotection. We hypothesized that at these time points CS in the supernatants were too diluted for a reliable quantification (the supernatants were completely refreshed at each time point and we analyzed 50 µL out of about 2.5 mL). Although further optimization is required, our results highlight the feasibility of the proposed approach.

## 5. Conclusions

In this study, we exploited CS polysaccharides produced by chemical modification of microbial-sourced unsulfated chondroitin. Since current pharmaceutical- and nutraceutical-grade CS derive from animal sources, their industrial production has to handle several safety, ecological, and economic issues, including numerous purification steps to remove all the contaminants. Therefore, the application of microbiological-chemical sourced CS polysaccharides could be rather convenient. After demonstrating that our CS-A,C and CS-A,C, K, L protect neuroblastoma SH-SY5Y cells against oxidative stress, we focused on the development of release systems based on Carbopol^®^ 974P NF and agarose. Our results highlight the potential of microbiological-chemical sourced CS against oxidative stress, a key condition of neurodegeneration, thus paving the way to the clinical translation of the therapeutic potential of such CS polysaccharides of non-animal origin.

## Data Availability

The data presented in this study are available in article.

## References

[1] Bedini E., Corsaro M.M., Fernández-Mayoralas A., Iadonisi A., Cohen E., Merzendorfer H. (2019). Chondroitin, dermatan, heparan, and keratan sulfate: Structure and functions. Extracellular Sugar-Based Biopolymers Matrices.

[2] Soares da Costa D., Reis R.L., Pashkuleva I. (2018). Sulfation of glycosaminoglycans and its implications in human health and disorders. Annu. Rev. Biomed. Eng..

[3] Couchman J.R., Pataki C.A. (2012). An Introduction to Proteoglycans and Their Localization. J. Histochem. Cytochem..

[4] Deepa S.S., Carulli D., Galtrey C., Rhodes K., Fukuda J., Mikami T., Sugahara K., Fawcett J.W. (2006). Composition of perineuronal net extracellular matrix in rat brain: A different disaccharide composition for the net-associated proteoglycans. J. Biol. Chem..

[5] Fawcett J.W., Oohashi T., Pizzorusso T. (2019). The roles of perineuronal nets and the perinodal extracellular matrix in neuronal function. Nat. Rev. Neurosci..

[6] Gama C.I., Tully S.E., Sotogaku N., Clark P.M., Rawat M., Vaidehi N., Goddard W.A., Nishi A., Hsieh-Wilson L.C. (2006). Sulfation patterns of glycosaminoglycans encode molecular recognition and activity. Nat. Chem. Biol..

[7] Carulli D., Pizzorusso T., Kwok J.C.F., Putignano E., Poli A., Forostyak S., Andrews M.R., Deepa S.S., Glant T.T., Fawcett J.W. (2010). Animals lacking link protein have attenuated perineuronal nets and persistent plasticity. Brain.

[8] Bruckner G., Hausen D., Hartig W., Drlicek M., Arendt T., Brauer K. (1999). Cortical areas abundant in extracellular matrix chondroitin sulphate proteoglycans are less affected by cytoskeletal changes in Alzheimer’s disease. Neuroscience.

[9] Morawski M., Bruckner M.K., Riederer P., Bruckner G., Arendt T. (2004). Perineuronal nets potentially protect against oxidative stress. Exp. Neurol..

[10] Sato Y., Nakanishi K., Tokita Y., Kakizawa H., Ida M., Maeda H., Matsui F., Aono S., Saito A., Kuroda Y. (2008). A highly sulfated chondroitin sulfate preparation, CS-E, prevents excitatory amino acid-induced neuronal cell death. J. Neurochem..

[11] Morawski M., Brückner G., Jäger C., Seeger G., Matthews R.T., Arendt T. (2012). Involvement of perineuronal and perisynaptic extracellular matrix in Alzheimer’s disease neuropathology. Brain Pathol..

[12] Baig S., Wilcock G.K., Love S. (2005). Loss of perineuronal net N-acetylgalactosamine in Alzheimer’s disease. Acta Neuropathol..

[13] Egea J., García A.G., Verges J., Montell E., López M.G. (2010). Antioxidant, antiinflammatory and neuroprotective actions of chondroitin sulfate and proteoglycans. Osteoarthr. Cartil..

[14] Mishra S., Ganguli M. (2021). Functions of, and replenishment strategies for, chondroitin sulfate in the human body. Drug Discov. Today.

[15] Iannuzzi C., Borriello M., D’Agostino A., Cimini D., Schiraldi C., Sirangelo I. (2019). Protective effect of extractive and biotechnological chondroitin in insulin amyloid and advanced glycation end product-induced toxicity. J. Cell. Physiol..

[16] Cañas N., Valero T., Villarroya M., Montell E., Vergés J., García A.G., López M.G. (2007). Chondroitin sulfate protects SH-SY5Y cells from oxidative stress by inducing Heme Oxygenase-1 via phosphatidylinositol 3-kinase/Akt. J. Pharmacol. Exp. Ther..

[17] Bravo R., Arimon M., Valle-Delgado J.J., García R., Durany N., Castel S., Cruz M., Ventura S., Fernàndez-Busquets X. (2008). Sulfated polysaccharides promote the assembly of amyloid beta(1-42) peptide into stable fibrils of reduced cytotoxicity. J. Biol. Chem..

[18] Zhao N., Meng J., Jiang W., Xu W., Liu C., Wang F. (2020). Study on the relationships between molecular weights of chondroitin sulfate oligosaccharides and Aβ-induced oxidative stress and the related mechanisms. Glycobiology.

[19] Köwitsch A., Zhou G., Groth T. (2018). Medical application of glycosaminoglycans: A review. J. Tissue Eng. Regen. Med..

[20] Guerrini M., Beccati D., Shriver Z., Naggi A., Viswanathan K., Bisio A., Capila I., Lansing J.C., Guglieri S., Fraser B. (2008). Oversulfated chondroitin sulfate is a contaminant in heparin associated with adverse clinical events. Nat. Biotechnol..

[21] Badri A., Williams A., Linhardt R.J., Koffas M.A.G. (2018). The road to animal-free glycosaminoglycan production: Current efforts and bottlenecks. Curr. Opin. Biotechnol..

[22] Badri A., Williams A., Awofiranye A., Datta P., Xia K., He W., Fraser K., Dordick J.S., Linhardt R.J., Koffas M.A.G. (2021). Complete biosynthesis of a sulfated chondroitin in *Escherichia coli*. Nat. Commun..

[23] Jin X., Zhang W., Wang Y., Sheng J., Xu R., Li J., Du G., Kang Z. (2021). Biosynthesis of non-animal chondroitin sulfate from methanol using genetically engineered *Pichia pastoris*. Green Chem..

[24] Bedini E., Laezza A., Iadonisi A. (2016). Chemical derivatization of sulfated glycosaminoglycans. Eur. J. Org. Chem..

[25] Corsuto L., Rother S., Koehler L., Bedini E., Moeller S., Schnabelrauch M., Hintze V., Schiraldi C., Scharnweber D. (2018). Sulfation degree not origin of chondroitin sulfate derivatives modulates keratinocyte response. Carbohydr. Polym..

[26] Vessella G., Vázquez J.A., Valcárcel J., Lagartera L., Monterrey D.T., Bastida A., García-Junceda E., Bedini E., Fernández-Mayoralas A., Revuelta J. (2021). Deciphering structural determinants in chondroitin sulfate binding to FGF-2: Paving the way to enhanced predictability of their biological functions. Polymers.

[27] Xiao Y., Li P., Cheng Y., Zhang X., Sheng J., Wang D., Li J., Zhang Q., Zhong C., Cao R. (2014). Enhancing the intestinal absorption of low molecular weight chondroitin sulfate by conjugation with α-linolenic acid and the transport mechanism of the conjugates. Int. J. Pharm..

[28] Kubo M., Ando K., Mimura T., Matsusue Y., Mori K. (2009). Chondroitin sulfate for the treatment of hip and knee osteoarthritis: Current status and future trends. Life Sci..

[29] Henrotin Y., Mathy M., Sanchez C., Lambert C. (2010). Chondroitin sulfate in the treatment of osteoarthritis: From in vitro studies to clinical recommendations. Ther. Adv. Musculoskelet. Dis..

[30] Hui J.H., Chan S.-W., Li J., Goh J.C.H., Li L., Ren X.F., Lee E.H. (2007). Intra-articular delivery of chondroitin sulfate for the treatment of joint defects in rabbit model. J. Mol. Histol..

[31] He Z., Wang B., Hu C., Zhao J. (2017). An overview of hydrogel-based intra-articular drug delivery for the treatment of osteoarthritis. Colloids Surf. B Biointerfaces.

[32] Dragicevic N., Krajisnik D., Milic J., Fahr A., Maibach H. (2019). Development of hydrophilic gels containing coenzyme Q10-loaded liposomes: Characterization, stability and rheology measurements. Drug Dev. Ind. Pharm..

[33] Al-Kinani A.A., Zidan G., Elsaid N., Seyfoddin A., Alani A.W., Alany R.G. (2018). Ophthalmic gels: Past, present and future. Adv. Drug Deliv. Rev..

[34] Benigni M., Pescina S., Grimaudo M.A., Padula C., Santi P., Nicoli S. (2018). Development of microemulsions of suitable viscosity for cyclosporine skin delivery. Int. J. Pharm..

[35] Mirza M.A., Panda A.K., Asif S., Verma D., Talegaonkar S., Manzoor N., Khan A., Ahmed F.J., Dudeja M., Iqbal Z. (2016). A vaginal drug delivery model. Drug Deliv..

[36] Kumria R., Nair A.B., Goomber G., Gupta S. (2016). Buccal films of prednisolone with enhanced bioavailability. Drug Deliv..

[37] Varum F.J.O., Veiga F., Sousa J.S., Basit A.W. (2011). Mucoadhesive platforms for targeted delivery to the colon. Int. J. Pharm..

[38] Jiang L., Gao L., Wang X., Tang L., Ma J. (2010). The application of mucoadhesive polymers in nasal drug delivery. Drug Dev. Ind. Pharm..

[39] Brady J., Dürig T., Lee P.I., Li J.-X., Qiu Y., Chen Y., Zhang G.G., Yu L., Mantri R.V. (2017). Polymer Properties and Characterization. Developing Solid Oral Dosage Forms: Pharmaceutical Theory and Practice.

[40] Liu J., Li L. (2005). SDS-aided immobilization and controlled release of camptothecin from agarose hydrogel. Eur. J. Pharm. Sci..

[41] Gustavsson E., Larsson O., Švec F., Tennikova T.B., Deyl Z. (2003). Chapter 6-Monolithic Polysaccharide Materials. Monolithic Materials-Preparation, Properties and Applications.

[42] Tunesi M., Prina E., Munarin F., Rodilossi S., Albani D., Petrini P., Giordano C. (2015). Cross-linked poly(acrylic acids) microgels and agarose as semi-interpenetrating networks for resveratrol release. J. Mater. Sci. Mater. Electron..

[43] Intagliata S., Modica M.N., Santagati L.M., Montenegro L. (2019). Strategies to Improve Resveratrol Systemic and Topical Bioavailability: An Update. Antioxidants.

[44] Tunesi M., Raimondi I., Russo T., Colombo L., Micotti E., Brandi E., Cappelletti P., Cigada A., Negro A., Ambrosio L. (2019). Hydrogel-based delivery of Tat-fused protein Hsp70 protects dopaminergic cells in vitro and in a mouse model of Parkinson’s disease. NPG Asia Mater..

[45] Dragan E.S. (2014). Design and applications of interpenetrating polymer network hydrogels. A review. Chem. Eng. J..

[46] Runge M.B., Wang H., Spinner R.J., Windebank A.J., Yaszemski M.J. (2012). Reformulating polycaprolactone fumarate to eliminate toxic diethylene glycol: Effects of polymeric branching andautoclave sterilization on material properties. Acta Biomater..

[47] Cimini D., De Rosa M., Carlino E., Ruggiero A., Schiraldi C. (2013). Homologous overexpression of *RfaH* in *E. coli* K4 improves the production of chondroitin-like capsular polysaccharide. Microb. Cell Factories.

[48] Bedini E., De Castro C., De Rosa M., Di Nola A., Iadonisi A., Restaino O.F., Schiraldi C., Parrilli M. (2011). microbiological-chemical strategy to produce chondroitin sulfate A,C. Angew. Chem. Int. Ed..

[49] Laezza A., De Castro C., Parrilli M., Bedini E. (2014). Inter vs. intraglycosidic acetal linkages control sulfation pattern in semi-synthetic chondroitin sulfate. Carbohydr. Polym..

[50] Rutala W.A., Weber D.J., Mandell G.L., Bennett J.E. (2015). Disinfection, Sterilization, and Control of Hospital Waste. Principles and Practice of Infectious Diseases.

[51] Winter S., Smith A., Lappin D., McDonagh G., Kirk B. (2017). Investigating steam penetration using thermometric methods in dental handpieces with narrow internal lumens during sterilizing processes with non-vacuum or vacuum processes. J. Hosp. Infect..

[52] Sasaki J.-I., Imazato S. (2020). Autoclave sterilization of dental handpieces: A literature review. J. Prosthodont. Res..

[53] Farndale R.W., Buttle D.J., Barrett A.J. (1986). Improved quantitation and discrimination of sulphated glycosaminoglycans by use of dimethylmethylene blue. Biochim. Biophys. Acta BBA Gen. Subj..

[54] Ransy C., Vaz C., Lombès A., Bouillaud F. (2020). Use of H_2_O_2_ to Cause Oxidative Stress, the Catalase Issue. Int. J. Mol. Sci..

[55] Farndale R.W., Sayers C.A., Barrett A.J. (1982). A Direct Spectrophotometric Microassay for Sulfated Glycosaminoglycans in Cartilage Cultures. Connect. Tissue Res..

[56] Ritger P.L., Peppas N.A. (1987). A simple equation for description of solute release II. Fickian and anomalous release from swellable devices. J. Control. Release.

[57] Thalla P.K., Fadlallah H., Liberelle B., Lequoy P., De Crescenzo G., Merhi Y., Lerouge S. (2014). Chondroitin sulfate coatings display low platelet but high endothelial cell adhesive properties favorable for vascular implants. Biomacromolecules.

[58] Förster Y., Schulze S., Penk A., Neuber C., Möller S., Hintze V., Scharnweber D., Schnabelrauch M., Pietzsch J., Huster D. (2020). The influence of different artificial extracellular matrix implant coatings on the regeneration of a critical size femur defect in rats. Mater. Sci. Eng. C.

[59] Ye J., Huang B., Gong P. (2021). Nerve growth factor-chondroitin sulfate/hydroxyapatite-coating composite implant induces early osseointegration and nerve regeneration of peri-implant tissues in Beagle dogs. J. Orthop. Surg. Res..

[60] Betancur M.I., Mason H.D., Alvarado-Velez M., Holmes P.V., Bellamkonda R.V., Karumbaiah L. (2017). Chondroitin Sulfate Glycosaminoglycan Matrices Promote Neural Stem Cell Maintenance and Neuroprotection Post-Traumatic Brain Injury. ACS Biomater. Sci. Eng..

[61] Altgärde N., Nilebäck E., de Battice L., Pashkuleva I., Reis R.L., Becher J., Möller S., Schnabelrauch M., Svedhem S. (2013). Probing the biofunctionality of biotinylated hyaluronan and chondroitin sulfate by hyaluronidase degradation and aggrecan interaction. Acta Biomater..

[62] Soleman S., Filippov M.A., Dityatev A., Fawcett J.W. (2013). Targeting the neural extracellular matrix in neurological disorders. Neuroscience.

[63] Miyata S., Kitagawa H. (2016). Chondroitin sulfate and neuronal disorders. Front. Biosci..

[64] Ju C., Gao J., Hou L., Wang L., Zhang F., Sun F., Zhang T., Xu P., Shi Z., Hu F. (2017). Neuroprotective effect of chondroitin sulfate on SH-SY5Y cells overexpressing wild-type or A53T mutant α-synuclein. Mol. Med. Rep..

[65] Yang X. (2020). Chondroitin sulfate proteoglycans: Key modulators of neuronal plasticity, long-term memory, neurodegenerative, and psychiatric disorders. Rev. Neurosci..

[66] McKeon R.J., Schreiber R.C., Rudge J.S., Silver J. (1991). Reduction of neurite outgrowth in a model of glial scarring following CNS injury is correlated with the expression of inhibitory molecules on reactive astrocytes. J. Neurosci..

[67] Muir E., De Winter F., Verhaagen J., Fawcett J. (2019). Recent advances in the therapeutic uses of chondroitinase ABC. Exp. Neurol..

[68] Vessella G., Traboni S., Cimini D., Iadonisi A., Schiraldi C., Bedini E. (2019). Development of semisynthetic, regioselective pathways for accessing the missing sulfation patterns of chondroitin sulfate. Biomacromolecules.

[69] Ma Q., Dudas B., Hejna M., Cornelli U., Lee J.M., Lorens S., Mervis R., Hanin I., Fareed J. (2002). The blood-brain barrier accessibility of a heparin-derived oligosaccharides C3. Thromb. Res..

[70] Galante R., Pinto T.J.A., Colaço R., Serro A.P. (2018). Sterilization of hydrogels for biomedical applications: A review. J. Biomed. Mater. Res. Part B Appl. Biomater..

[71] Pugliese A., Toresco M., McNamara D., Iuga D., Abraham A., Tobyn A., Hawarden L.E., Blanc F. (2021). Drug-Polymer Interactions in Acetaminophen/Hydroxypropylmethylcellulose Acetyl Succinate Amorphous Solid Dispersions Revealed by Multidimensional Multinuclear Solid-State NMR Spectroscopy. Mol. Pharm..

